# Aberrant White Matter Development in Cerebral Visual Impairment: A Proposed Mechanism for Visual Dysfunction Following Early Brain Injury

**DOI:** 10.31083/j.jin2301001

**Published:** 2024-01-10

**Authors:** Corinna M. Bauer, Lotfi B. Merabet

**Affiliations:** 1 Laboratory for Visual Neuroplasticity, Department of Ophthalmology, Massachusetts Eye and Ear Infirmary, Harvard Medical School, Boston, MA 02114, USA; 2 Lab of Neuroimaging and Vision Science, Gordon Center for Medical Imaging, Department of Radiology, Massachusetts General Hospital, Harvard Medical School, Boston, MA 02114, USA

**Keywords:** cerebral visual impairment, cortical visual impairment, tractography, dorsal visual stream, ventral visual stream, periventricular leukomalacia

## Abstract

**Background:**

Cerebral visual impairment (CVI) is a common sequala of early brain injury, damage, or malformation and is one of the leading individual causes of visual dysfunction in pediatric populations worldwide. Although patients with CVI are heterogeneous both in terms of underlying etiology and visual behavioural manifestations, there may be underlying similarities in terms of which white matter pathways are potentially altered. This exploratory study used diffusion tractography to examine potential differences in volume, quantitative anisotropy (QA), as well as mean, axial, and radial diffusivities (mean diffusivity (MD), axial diffusivity (AD) and radial diffusivity (RD), respectively) focusing on the dorsal and ventral visual stream pathways in a cohort of young adults with CVI compared to typically sighted and developing controls.

**Methods:**

High angular resolution diffusion imaging (HARDI) data were acquired in a sample of 10 individuals with a diagnosis of CVI (mean age = 17.3 years, 2.97 standard deviation (SD), range 14–22 years) and 17 controls (mean age = 19.82 years, 3.34 SD, range 15–25 years). The inferior longitudinal fasciculus (ILF), inferior fronto-occipital fasciculus (IFOF), vertical occipital fasciculus (VOF), and the three divisions of the superior longitudinal fasciculus (SLF I, II, and III) were virtually reconstructed and average tract volume (adjusted for intracranial volume), MD, AD, and RD were compared between CVI and control groups. As a secondary analysis, an analysis of variance (ANOVA) was carried out to investigate potential differences based on etiology (i.e., CVI due to periventricular leukomalacia (CVI-PVL) and CVI due to other causes (CVI-nonPVL)).

**Results:**

We observed a large degree of variation within the CVI group, which minimized the overall group differences in tractography outcomes when examining the CVI sample as a unitary group. In our secondary analysis, we observed significant reductions in tract volume in the CVI-PVL group compared to both controls and individuals with CVI due to other causes. We also observed widespread significant increases in QA, MD, and AD in CVI-PVL compared to the control group, with mixed effects in the CVI-nonPVL group.

**Conclusions:**

These data provide preliminary evidence for aberrant development of key white matter fasciculi implicated in visual perceptual processing skills, which are often impaired to varying degrees in individuals with CVI. The results also indicate that the severity and extent of the white matter changes may be due in part to the underlying cause of the cerebral visual impairments. Additional analyses will need to be done in a larger sample alongside behavioural testing to fully appreciate the relationships between white matter integrity, visual dysfunction, and associated causes in individuals with CVI.

## 1. Introduction

Individuals with cerebral visual impairment (CVI)—one of the foremost individual causes of pediatric visual impairment in many countries around the world [1–6]—often present with a complex amalgam of visual dysfunctions. These may include impairments in visual function, such as reduced visual acuity, restricted visual fields, abnormal eye movements, and reduced contrast sensitivity, as well as impairments in visuo-perceptual tasks associated with the dorsal and ventral visual streams, including (but not limited to) visual attention, processing moving stimuli, object recognition, and face recognition [7–12].

Previous research using neuroimaging and animal models indicates that many dorsal stream functions are primarily processed within the occipital and parietal cortices with additional involvement of the frontal cortex. These regions are anatomically connected via the superior longitudinal fasciculus (SLF) and inferior fronto-occipital fasciculus (IFOF), in addition to local U-fibers [13–16]. Indeed, injury to these white matter pathways often results in impaired dorsal stream functions [17–20]. On the other hand, the ventral stream functions are focused in the occipitotemporal regions, which are primarily connected via the inferior longitudinal fasciculus (ILF). Similarly, injury to the ILF is often associated with impaired face and object recognition [21,22]. In recent years there has also been increasing recognition of the communication between the dorsal and ventral streams [23–25]. Although the precise anatomical correlate of this has yet to be agreed upon, one of the main direct pathways that is likely is the vertical occipital fasciculus (VOF), which connects the dorsal and ventral aspects of the occipital lobe [24]. While integrity of these pathways has previously been linked with visual perceptual abilities in CVI [22,26,27], an in-depth analysis of the white matter tracts associated with the dorsal and ventral streams has yet to be reported in CVI.

This study used diffusion tractography derived from high angular resolution diffusion imaging (HARDI) data to investigate the volume and integrity (including anisotropy and diffusivities) of the white matter pathways of the dorsal and ventral visual streams in CVI compared to typically sighted and developing controls adjusting for the potential effects of age. Additional exploratory analyses were conducted to (1) investigate the potential differential impact of CVI on development of the subdivisions of the SLF, and (2) start to determine if white matter integrity is different based on the specific etiology of CVI. We were specifically interested in whether those with CVI due to periventricular leukomalacia (PVL) demonstrated more severe white matter injury than those with CVI due to other causes.

## 2. Materials and Methods

### 2.1 Participants

Twenty-seven participants, 10 individuals with CVI (mean age = 17.3 years, 2.97 standard deviation (SD), range 14–22 years) and 17 controls (mean age = 19.82 years, 3.34 SD, range 15–25 years), completed this study. A full list of participant details, including suspected causes of CVI can be found in Table 1. Control participants had normal or corrected-to-normal visual acuity with no history of neurologic or vision disorders. All participants with CVI were previously diagnosed by their eyecare and vision professionals who have extensive clinical expertise working with this population [11]. Specifically, the diagnosis was based on directed and objective assessments of visual functions including acuity, contrast, visual fields, colour, and oculomotor functions, in addition to a thorough refractive and ocular examination. Functional vision was also evaluated using a variety of methods including structured questionnaires, surveys, and activities instigating how the individual uses their vision during different tasks. Thus, the diagnosis of CVI was based on an integrated review of medical history and available neuroimaging and electrophysiology records. In each case, the level of functional visual impairment was beyond what was expected based on visual function alone and could not be attributed to any potentially co-occurring ocular or cognitive condition. Note that the underlying etiology associated with CVI was diverse, including PVL, seizure disorder, neonatal infection, anoxia, and pre-eclampsia. Because four participants had the same underlying etiology (i.e., PVL), as an exploratory analysis we sub-divided the CVI group into those with CVI due to periventricular leukomalacia (CVI-PVL) and those with CVI due to other causes (CVI-nonPVL).

The study was approved by the investigative review board of the Massachusetts Eye and Ear, Boston, MA, USA (IRB number 2019P003229). Written informed consent (assent in the case of minors) was obtained from all participants (and their parents) prior to participation in the study.

### 2.2 MRI Acquisition and Analysis

Magnetic resonance imaging (MRI) data were acquired on a 3T Philips Achieva System (Philips, Best, the Netherlands) with an 8-channel phased array head coil. Two structural T_1_-weighted scans were acquired using a turbo spin echo sequence (echo time (TE) = 3.1 ms, relaxation time (TR) = 6.8 ms, flip angle = 9°, voxel size 0.98 × 0.98 × 1.20 mm) and HARDI and accompanying field maps were acquired with a single-shot EPI sequence (TE = 73 ms, TR = 17,844 ms, flip angle 90°, 64 directions, EPI factor = 59, Bmin = 0 s/mm^2^, Bmax = 3000 s/mm^2^, voxel size = 2 mm isotropic, enhanced gradients at 80 mT/m, and a slew rate of 100 T/m/ms).

Similar to our previous work [28], HARDI data were skull-stripped and corrected for motion and eddy currents in FSL 5.0.8 (FMRIB Software Library, http://fsl.fMRib.ox.ac.uk/fsl). The orientation distribution function (ODF) was reconstructed in DSI-Studio (http://dsi-studio.labsolver.org) using generalized q-sampling imaging [29] with a diffusion sampling length ratio of 1.25, ODF sharpening via decomposition [30], decomposition fraction of 0.04, and maximum fiber population of 8. Three fibers per voxel were resolved with an 8-fold ODF tessellation. HARDI data were co-registered to the corresponding T_1_-weighted structural image for each subject using boundary-based registration in FreeSurfer 6.0.1 (http://surfer.nmr.mgh.harvard.edu/) [31]. Registration accuracy was verified for each subject and manual corrections were performed where necessary. Each of the 68 cortical T_1_-weighted parcellations [32] were reverse transformed into subject-specific HARDI space, creating the seed (start) and target (end) point regions of interests (ROIs) for tractography analysis.

An in-house MATLAB script utilizing the tractography functions from DSI-Studio generated streamlines between each set of ROIs (see below). Tractography parameters were as follows: termination angle = 45°, subject-specific quantitative anisotropy (QA) threshold [33,34], smoothing = 0.5, step size = 0.5 mm, minimum length = 5 mm, maximum length = 300 mm, random fiber direction, Gaussian radial interpolation, and 100,000 seeds. Tract volume and mean fractional anisotropy (FA), QA, axial diffusivity (AD), radial diffusivity (RD), and mean diffusivity (MD) were examined for each reconstructed fasciculus bilaterally.

#### 2.2.1 Parcellation of Fasciculi

**Fig. 1.**
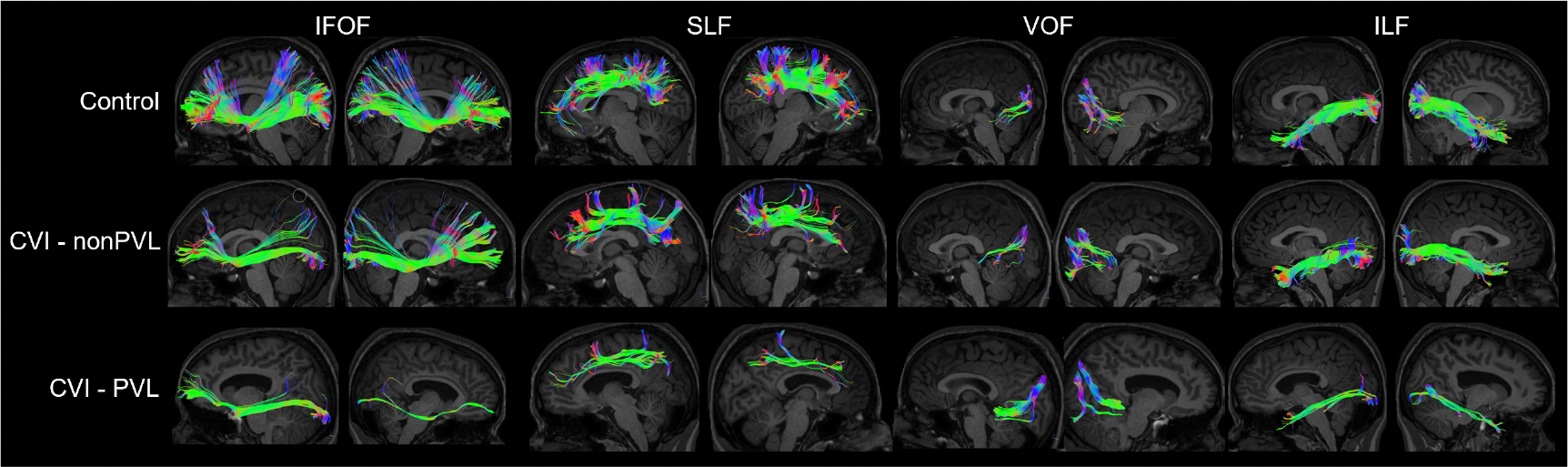
Representative reconstructions of the IFOF, SLF, VOF, and ILF for control (top), CVI due to other causes (CVI-nonPVL) (birth complications-CVI participant 4, middle), and CVI-PVL (CVI participant 3, bottom) participants. Note the enlarged ventricles present in the particpant with CVI-PVL. IFOF, inferior fronto-occipital fasciculus; SLF, superior longitudinal fasciculus; VOF, vertical occipital fasciculus; ILF, inferior longitudinal fasciculus.

All fasciculi were defined according to the start and end points bidirectionally derived from anatomical and tractography literature as outlined below. Each tract was visually assessed for accuracy. Representative reconstructions for control, CVI-PVL, and CVI-nonPVL participants are shown in Fig. 1.

For the SLF, start points were in the cuneus, interior parietal lobe, lateral occipital cortex, pericalcarine, precuneus, superior parietal lobe, and supramarginal gyrus. End points were in the caudal middle frontal, lateral orbitofrontal, medial orbitofrontal, pars opercularis, pars orbitalis, pars triangularis, rostral middle frontal, and superior frontal regions [15,16,35–38]. This enabled the reconstruction of SLF divisions I, II, and III which were combined to generate a global SLF. Any tracts that did not belong to one of the three main divisions of the SLF (i.e., those which traversed inferiorly representing the IFOF, those which crossed the midline, and those within the cingulate cortex) were manually removed.

For the IFOF, start points were located in the cuneus, fusiform gyrus, inferior parietal lobe, lateral occipital cortex (specifically the superior, inferior, and middle occipital gyri), lingual gyrus, pericalcarine, precuneus, and superior parietal lobe. End points were located in the caudal middle frontal gyrus, lateral and medial orbitofrontal gyri, pars opercularis, orbitalis, and triangularis, rostral middle frontal, superior frontal gyrus, and frontal pole [13,14,39–42]. Any tracts which did not pass through the ventral aspect of the extreme and external capsules were removed as were those belonging to the SLF or crossing the midline.

For the ILF, start points were defined in the cuneus, lingual gyrus, pericalcarine, lateral occipital, and inferior occipital gyrus. End points were defined in the fusiform gyrus, inferior, middle, and superior temporal gyri, as well as the temporal pole [21,43–48]. This enabled the reconstruction of the fusiform, dorsolateral, lingual, and cuneal branches of the ILF. The ILF traverses infero-lateral to the optic radiations and ventricles and originated on the lateral-inferior wall of the occipital horn. As such, any tracts belonging to the VOF, short U-fibres, or the middle longitudinal fasciculus were excluded.

For the VOF, start and end points were defined in the cuneus, fusiform gyrus, lingual gyrus, lateral occipital cortex, and parahippocampal gyrus and were restricted to the tracts connecting the superior and inferior portions of the occipital lobe. Short U-fibres were also excluded [24,46,49–52].

### 2.3 Statistical Methods

Potential differences in age between control and CVI groups were evaluated using Student’s *t*-test, while differences between control PVL, and CVI-nonPVL were investigated with an analysis of variance (ANOVA). Chi-square was used to investigate distribution of males and females between groups.

As a first level, we investigated differences in tractography outcomes (i.e., volume, QA, FA, AD, RD, and MD) between CVI and control groups. Because there was a significant age differences between the groups and because white matter continues to develop into the fourth decade of life [53], age in years was entered as a covariate in the analysis. The potential interaction between group and age was included as an initial term in the statistical models. All analyses involving tract volume were first adjusted for individual intracranial volume using residuals. Significance level was set at *p* < 0.05. Bonferroni correction was applied at *p* < 0.001 (e.g., *p* = 0.05/48; 4 tracts per hemisphere * 2 hemispheres * 6 measures per tract).

As a separate analysis, we also subdivided the SLF into its three main divisions and investigated group differences within each. Bonferroni correction for the SLF analysis was applied at *p* < 0.0014 (e.g., *p* = 0.05/36 (3 tracts per hemisphere * 2 hemispheres * 6 measures per subdivision of the SLF).

As an exploratory analysis, we investigated the potential impact of CVI etiology, specifically evaluating whether there is a differential impact of PVL (CVI-PVL) as compared to other causes of CVI (CVI-nonPVL). Significance level was set at *p* < 0.05 with post-hoc correction for multiple comparisons.

## 3. Results

A small but significant difference in age between CVI and control groups was observed (mean age CVI = 17.3 years, 2.97 SD, range 14–22 years; mean age controls = 19.82 years, 3.34 SD, range 15–25 years; t (25) = 2.07, *p* = 0.049). When the CVI group was divided by etiology (i.e., PVL, n = 4 and CVI-nonPVL, n = 6), no significant differences in age were observed (F = 3.31, *p* = 0.054). Males and females were equally distributed between CVI and control groups (Chi-square = 2.44, *p* = 0.12).

### 3.1 Tract Differences between CVI and Control Groups Adjusting for Age

#### 3.1.1 Tract Volume Adjusting for Intracranial Volume (ICV)

No significant difference in volume between control and CVI groups were observed for any of the reconstructed tracts after adjusting for age and intracranial volume. A significant age effect was observed for volume of the right SLF II (F = 7.06, *p* = 0.01), however this did not survive correction for multiple comparisons. No other age effects were observed for tract volume (Table 2, Supplementary Table 1, Supplementary Fig. 1).

#### 3.1.2 Quantitative Anisotropy

Overall, there were no significant differences in QA between groups and there were also no significant effects of age for any of the measured tracts (Table 3, Supplementary Table 1).

#### 3.1.3 Mean Diffusivity

**Fig. 2.**
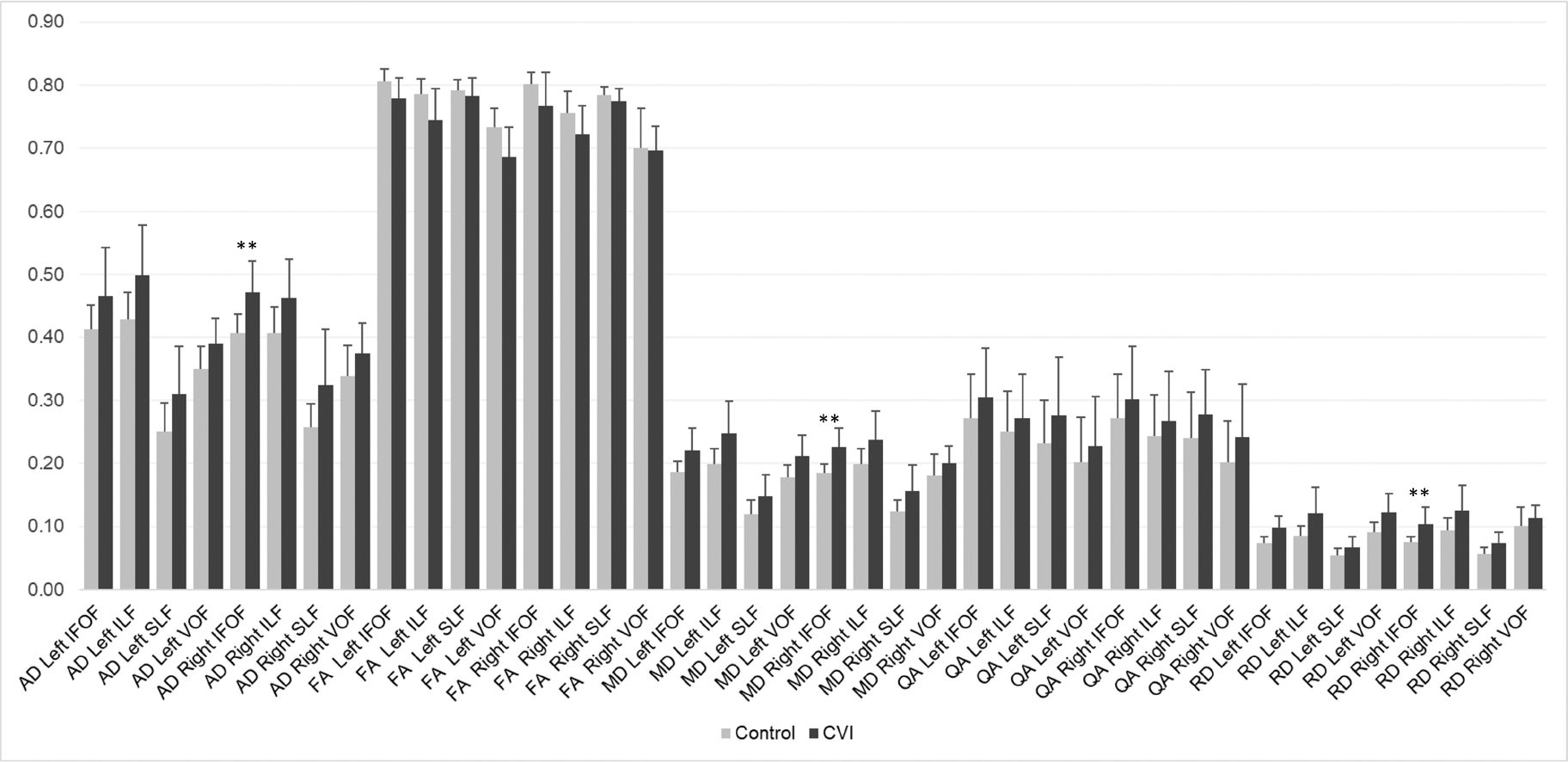
Group comparisons in diffusivity and anisotropy measures adjusted for age (ANCOVA). ** *p* < 0.05 following Bonferroni correction for multiple comparisons. AD, axial diffusivity; FA, fractional anisotropy; MD, mean diffusivity; QA, quantitative anisotropy; RD, radial diffusivity.

Adjusting for the potential effects of age, we observed a significant overall effect of group for MD of the left IFOF (F = 8.86, *p* = 0.0067), ILF (F = 11.1, *p* = 0.0028), SLF (F = 5.44, *p* = 0.0284), and VOF (F = 9.42, *p* = 0.0053), as well as the right IFOF (F = 23.76, *p* < 0.0001), ILF (F = 11.85, *p* = 0.0021), and SLF (F = 8.44, *p* = 0.0078) (Table 4). Of these, only MD of the right IFOF survived correction for multiple comparisons. Post-hoc analysis revealed that the MD in the right IFOF was significantly higher for the CVI group compared to controls (adjusted mean CVI = 0.229, adjusted mean control = 0.184, *p* < 0.0001) (Fig. 2).

In our analysis of SLF subdivisions, we observed a significant overall increase in MD in CVI compared to controls for the left SLF I (F = 9.84, *p* = 0.0045) and SLF II (F = 7.07, *p* = 0.0147), as well as the right SLF I (F = 6.5, *p* = 0.0176) and SLF III (F = 10.6, *p* = 0.0036). These did not survive correction for multiple comparisons (Supplementary Table 1).

There were no significant effects of age on MD for any of the tracts (Table 4).

#### 3.1.4 Axial Diffusivity

Adjusting for the potential effects of age, we observed a significant overall effect of group on AD of the left IFOF (R = 5.62, *p* = 0.0265), ILF (F = 9.34, *p* = 0.0054), SLF (F = 5.7, *p* = 0.0252), and VOF (F = 5.43, *p* = 0.0285); as well as the right IFOF (F = 16.95, *p* = 0.0004), ILF (F = 9.31, *p* = 0.0055), and SLF (F = 7.67, *p* = 0.0107) (Table 5). Of these, only AD of the right IFOF survived correction for multiple comparisons. Post-hoc analysis revealed that the AD in the right IFOF was significantly higher for the CVI group compared to controls (adjusted mean CVI = 0.473, adjusted mean control = 0.405, *p* = 0.0004) (Fig. 2).

In our analysis of SLF subdivisions, we observed a significant overall increases AD in CVI compared to controls for the left SLF I (F = 8.23, *p* = 0.0085) and SLF II (F = 6.41, *p* = 0.0194), as well as the right SLF I (F = 5.96, *p* = 0.0224) and SLF III (F = 9.85, *p* = 0.0048). These did not survive correction for multiple comparisons (Supplementary Table 1).

There were no significant effects of age on AD for any of the tracts (Table 5).

#### 3.1.5 Radial Diffusivity

Adjusting for the potential effects of age, a significant overall effect of group was observed for RD of the left IFOF (F = 11.51, *p* = 0.0025), ILF (F = 10.33, *p* = 0.0037), and VOF (F = 11.04, *p* = 0.0028); as well as the right IFOF (F = 17.27, *p* = 0.0003), ILF (F = 12.61, *p* = 0.0016), and SLF (F = 9.02, *p* = 0.0062) (Table 6). Of these, only RD of the right IFOF survived correction for multiple comparisons. Post-hoc analysis revealed that the RD in the right IFOF was significantly higher for the CVI group compared to controls (adjusted mean CVI = 0.106, adjusted mean control = 0.073, *p* = 0.0003) (Fig. 2).

In our analysis of SLF subdivisions, we observed a significant overall increased in RD in CVI compared to controls for the left SLF I (F = 5.59, *p* = 0.0265 and SLF II (F = 5.19, *p* = 0.0333), as well as the right SLF I (F = 6.04, *p* = 0.0216) and SLF III (F = 7.94, *p* = 0.01). These did not survive correction for multiple comparisons (Supplementary Table 1).

A significant age effect was observed for RD of the right ILF (F = 5.08, *p* = 0.0337), however this did not survive correction for multiple comparisons. No other age effects were observed for RD (Table 6).

#### 3.1.6 Mean Fractional Anisotropy

Adjusting for the potential effects of age, we observed a significant group difference in FA of the left ILF (F = 6.26, *p* = 0.0196) and VOF (F = 9.91, *p* = 0.0044), as well as the right IFOF (F = 8.07, *p* = 0.009) and ILF (F = 11.31, *p* = 0.0026). However, none of these survived Bonferroni correction for multiple comparisons (Table 7).

There were no significant differences in FA between CVI and control groups in our analysis of SLF subdivisions (Supplementary Table 1).

We observed a significant effect of age on FA of the right ILF (F = 7.47, *p* = 0.0116), however, this did not survive Bonferroni correction (Table 7).

### 3.2 Tract Differences between CVI-PVL, CVI-nonPVL, and Control Groups Adjusting for Age

As an exploratory analysis, we investigated the potential impact of the reported cause of CVI on markers of white matter tract integrity, specifically focusing on PVL compared to other nonPVL causes. Details can be found in Supplementary Tables 2–8.

#### 3.2.1 Tract Volume Adjusting for ICV

Adjusting for the potential effects of age, we observed a significant overall effect of CVI etiology on volume of the left ILF (F = 4.59, *p* = 0.021, post-hoc: CVI-PVL significantly smaller than both control and CVI-nonPVL groups), the right IFOF (F = 3.73, *p* = 0.039, post-hoc: CVI-PVL significantly smaller than either controls or CVI-nonPVL), right ILF (F = 4.49, *p* = 0.023, post-hoc: CVI-PVL significantly smaller than either controls or CVI-nonPVL), and right SLF (F = 3.78, *p* = 0.038, post-hoc: CVI-PVL significantly smaller than either controls or CVI-nonPVL).

A significant overall effect of age was observed for volume of the left IFOF (F = 4.71, *p* = 0.04, increasing volume with age), SLF (F = 5.65, *p* = 0.026, increasing volume with age), and SLF I (F = 5.35, *p* = 0.03, increasing volume with age); as well as the right SLF (F = 5.64, *p* = 0.026, increasing volume with age), and SLF II (F = 7.40, *p* = 0.013, increasing volume with age).

#### 3.2.2 Quantitative Anisotropy

Adjusting for the potential effects of age, we observed a significant overall effect of CVI etiology on QA of the left SLF II (F = 5.59, *p* = 0.012), SLF III (F = 7.51, *p* = 0.004). Specifically, those with PVL showed significantly higher QA in the left SLF II and III than either control participants or those with CVI due to other etiologies.

We observed a significant overall effect of age on QA of the right ILF (F = 5.43, *p* = 0.029) and right VOF (F = 4.91, *p* = 0.037), whereby QA tended to decrease across both groups with increasing age.

#### 3.2.3 Mean Diffusivity

Adjusting for the potential effects of age, we observed a significant overall effect of CVI etiology on MD of the left IFOF (F = 7.47, *p* = 0.0033, post-hoc: CVI-PVL significantly greater than control and CVI-nonPVL groups), ILF (F = 9.06, *p* = 0.0013, post-hoc: CVI-PVL significantly greater than control and CVI-nonPVL groups), SLF (F = 3.79, *p* = 0.038, post-hoc: CVI-PVL significantly greater than controls, but not CVI-nonPVL), SLF I (F = 7.82, *p* = 0.0026, post-hoc: CVI-PVL significantly greater than control and CVI-nonPVL groups), SLF II (F = 5.22, *p* = 0.015, post-hoc: CVI-PVL significantly greater than controls, but not CVI-nonPVL), and VOF (F = 4.82, *p* = 0.018, post-hoc: CVI-nonPVL significantly greater than the control group); as well as for the right IFOF (F = 17.79, *p* < 0.0001, post-hoc: CVI-PVL greater than both controls and CVI-nonPVL, and CVI-nonPVL greater than controls), ILF (F = 6.52, *p* = 0.0057, post-hoc: both CVI-PVL and CVI-nonPVL groups significantly greater than the control group), SLF (F = 8.76, *p* = 0.0015, post-hoc: CVI-PVL significantly greater than control and CVI-nonPVL groups), SLF I (F = 6.68, *p* = 0.0051, CVI-PVL significantly greater than control and CVI-nonPVL groups), and SLF III (F = 5.65, *p* = 0.011, post-hoc: CVI-PVL greater than control and CVI-nonPVL groups with an additional trend for CVI-nonPVL greater than controls (*p* = 0.051)).

There was also no significant overall effect of age for MD.

#### 3.2.4 Axial Diffusivity

Adjusting for the potential effects of age, we observed a significant overall effect of CVI etiology on AD of the left IFOF (F = 5.14, *p* = 0.015, post-hoc: CVI-PVL significantly greater than controls, with a trend for an increase compared to CVI-nonPVL ( *p* = 0.0598), ILF (F = 8.01, *p* = 0.0023, post-hoc: CVI-PVL greater than both controls and CVI-nonPVL CVI), SLF (F = 5.35, *p* = 0.012, post-hoc: CVI-PVL significantly greater than controls, with a trend for an increase compared to CVI-nonPVL ( *p* = 0.051)), SLF I (F = 8.84, *p* = 0.0014, post-hoc: CVI-PVL greater than both controls and CVI-nonPVL CVI), and SLF II (F = 7.45, *p* = 0.0038, post-hoc: CVI-PVL greater than both controls and CVI-nonPVL CVI); as well as the right IFOF (F = 15.84, *p* < 0.0001, post-hoc: CVI-PVL greater than both controls and CVI-nonPVL CVI, trend for CVI-nonPVL greater than controls (*p* = 0.053)), ILF (F = 5.11, *p* = 0.015, post-hoc: CVI-PVL significantly greater than controls, with a trend for CVI-nonPVL greater than controls (*p* = 0.07)), SLF (F = 9.76, *p* = 0.0009, post-hoc: CVI-PVL greater than both controls and CVI-nonPVL CVI), SLF I (F = 6.99, *p* = 0.0042, post-hoc: CVI-PVL greater than both controls and CVI-nonPVL CVI), and SLF III (F = 5.96, *p* = 0.0089, post-hoc: CVI-PVL significantly greater than controls, but not CVI-nonPVL).

There was no significant overall effect of age on AD.

#### 3.2.5 Radial Diffusivity

Adjusting for the potential effects of age, we observed a significant overall effect of CVI etiology on RD of the left IFOF (F = 8.09, *p* = 0.0023, post-hoc: CVI-PVL and CVI-nonPVL significantly greater than controls, with an additional trend for increased RD between CVI-PVL and CVI-nonPVL causes of CVI (*p* = 0.077)), ILF (F = 7.62, *p* = 0.0029, post-hoc: CVI-PVL significantly greater than controls and a trend for increase compared to the CVI-nonPVL group), and VOF (F = 5.83, *p* = 0.0090, post-hoc: CVI-nonPVL causes of CVI were significantly greater than controls, with a non-significant trend for increase compared to CVI-PVL (*p* = 0.057) and no differences between CVI-PVL and controls); as well as the right IFOF (F = 9.80, *p* = 0.0008, post-hoc: CVI due to CVI-PVL or CVI-nonPVL causes were significantly greater than controls), ILF (F = 6.90, *p* = 0.0045, post-hoc: both CVI-PVL and CVI-nonPVL causes of CVI were significantly greater than controls), SLF (F = 6.18, *p* = 0.0071, post-hoc: CVI-PVL significantly greater than controls), SLF I (F = 4.52, *p* = 0.022, post-hoc: CVI-PVL significantly greater than controls), and SLF III (F = 3.79, *p* = 0.029, post-hoc: CVI due to CVI-PVL or CVI-nonPVL causes were significantly greater than controls).

There was no significant overall effect of age on RD.

#### 3.2.6 Mean Fractional Anisotropy

Adjusting for the potential effects of age, we observed a significant overall effect of CVI etiology on FA of the left VOF (F = 5.02, *p* = 0.016, post-hoc: CVI-nonPVL was significantly reduced compared to controls, while there was a trend for reduced FA in the CVI-PVL group compared to controls (*p* = 0.057), right IFOF (F = 3.96, *p* = 0.033, post-hoc: CVI-PVL significantly lower than controls with a trend for CVI-nonPVL to be lower than controls (*p* = 0.055)), and right ILF (F = 5.82, *p* = 0.0090, post-hoc: CVI due to PVL and CVI-nonPVL etiologies were significantly lower than controls).

There was a significant overall effect of age on FA of the right ILF (F = 5.68, *p* = 0.0258, decreasing FA with age).

## 4. Discussion

This study sought to identify differences in white matter integrity in white matter tracts associated with the extended visual processing networks in a cohort of youths and young adults with CVI. In the main analysis, following correction for multiple comparisons we observed significant increases in mean, axial, and radial diffusivity of the right IFOF in CVI compared to controls. Additionally, two subanalyses were performed. First, the potential differences in tract integrity with CVI across the three divisions of the SLF were investigated. Second, the potential impact of CVI etiology (i.e., PVL as compared to non-PLV causes) on tract outcomes was investigated as an exploratory analysis.

The main findings of this study indicate that the right IFOF may be particularly impacted in CVI. The IFOF connects parieto-occipital regions with the lateral frontal cortex through the floor of the external capsule [35,41,54], although its precise functions remain unclear. Based on functional imaging and anatomical studies, it has been postulated that the IFOF may play a role in face recognition [55], multimodal sensory-motor integration [40], reading and writing [56,57], and the semantic processing of language [40,58,59], with the right IFOF potentially being involved in executive control [54,60] and memory [61]. Because CVI is often caused by early brain injury it is possible that the IFOF, being a long-range bundle, is particularly susceptible to disruption [62,63]. Moreover, individuals with CVI often demonstrate a complex of manifestations that may encompass not only visual functions like face recognition [7], but cognition, executive functioning, and other multisensory processes such as reading and writing [64], each of which may in part be subserved by the IFOF.

The underlying etiologies leading to CVI in our participants are diverse, including seizure disorder, PVL, neonatal stroke, genetic disorder, and birth trauma, making it challenging to interpret the complex changes in diffusion signal that were observed in this study across the entire CVI group. However, dysmyelination and white matter injury have been noted in a number of the represented etiologies, including periventricular leukomalacia [65–67], seizure disorder [68], and neonatal stroke [69]. Thus, it is a parsimonious explanation that the observed changes in diffusivity measures mainly reflect axonal injury and dysmyelination.

In this study we observed a significant increase in RD within the right IFOF in CVI compared to controls. Increased RD is associated with myelin damage (i.e., demyelination, abnormal myelin wrapping, etc.) [70,71] and has been observed in many neurodegenerative [72–75], movement [76], and other neurologic disorders [77]. However, changes in RD may not be specific to myelin integrity, but may also reflect changes in the extra-axonal water content [78,79]. Thus, care needs to be taken when interpreting changes in RD, particularly when the clinical population comprises mixed etiologies as is the case here [80].

Similarly, the interpretation of AD is complex. Although alterations in AD are thought to reflect axonal injury, the precise pathological changes associated with AD changes are unclear as they do not always correspond to the underlying pathology. Evidence from both animal models and demyelinating conditions like multiple sclerosis indicate that AD changes may vary as a function of the stage of axon injury and/or the underlying processes. Specifically increased AD may reflect axonal swelling, degeneration, or diffuse injury [81]. Additionally, during the early phases of white matter injury, AD may be decreased, corresponding to axonal loss [82]. Thus, there are various reasons which may underlie the observed increases in AD in this study. However, many of the individuals with CVI who participated in this study demonstrated diffusion white matter pathology as evidenced in their FLAIR scans (Supplementary Fig. 2). Additional white matter imaging methods and analysis techniques, such as myelin water fraction imaging [83,84] or neurite orientation dispersion and density imaging (NODDI) [85] may help characterize the underlying mechanisms of the observed white matter changes.

While we observed qualitative reductions in volume for a number of the tracts in CVI compared to controls, these did not reach statistical significane, likely due to the small sample size and heterogeneity with the group of participants with CVI. Our exploratory analysis investigating the impact of etiology revealed a significant reduction in volume only in the CVI-PVL group for the ILF, IFOF, and SLF; which suggests that there may be differential effects of etiology on the development of white matter pathways. However, this needs to be replicated in a larger sample.

Together, the observed changes in diffusivity measures suggest that there is likely a confluence of white matter abnormalities in the IFOF in this small heterogeneous sample of individuals with CVI. The results suggest some of that potential underlying changes include reduced myelination, myelin damage, and/or axonal loss. However, additional analyses using complementary techniques, such as myelin water imaging and multicompartment diffusion modelling, are needed to help differentiate these possibilities.

### 4.1 Exploratory Analysis on the Impact of Etiology

Preliminary exploratory analysis into potential impact of etiology on tractography outcomes suggests that certain etiologies of CVI, such as PVL, may be associated with more widespread and severe changes in tract integrity across multiple distributed white matter pathways. The exploratory analysis indicates that CVI caused by PVL may result in more substantial impairments in white matter myelination and integrity. This is in line with histological evidence suggesting impaired myelination due to a lack of mature myelin-producing oligodendrocytes [65,66,86,87]. However, caution should be taken when interpreting these findings due to the small sample size of this sub-analysis. A larger sample of individuals with CVI due to PVL as well as other causes is needed to better understand how the long-term neurodevelopmental ramifications of PVL differ from other etiologies of CVI. Further, the extent to which CVI contributes to the observed white matter changes beyond those seen in PVL (i.e., PVL without CVI) is unclear. In other words, it is unclear whether there are compounding effects of having both CVI and PVL diagnoses, as opposed to just PVL.

### 4.2 Limitations

Although efforts were made to proceed with a robust protocol, there are inherent limitations to this study. First, there is a limited sample size presented in this study and additional studies with a larger sample are needed to verify the results. Also, manual delineation of white matter tracts is inherently subjective and is challenging to complete in brains with grossly abnormal morphology (i.e., enlarged ventricles, focal lesions, etc.). To minimize these potential effects and increase consistency across subjects, a common protocol was applied for tractography across all participants. Future studies implementing automated tractography methods (e.g., [88–90] would be useful to confirm these findings. However, it remains unclear how these automated tractography algorithms perform in the case of early developmental brain injury, particularly those with visible malformations and widespread white matter lesions. Finally, the current study focused on investigating potential group differences in tract outcomes independent of level of visual dysfunction. Thus, it is not possible to draw any conclusions regarding the severity of the impact of CVIs on daily functioning or on the relationship between tract integrity and level of visual dysfunction in the CVI group. Additional studies correlating tract outcomes and visual ability are still needed to better understand this complex relationship. Notably, in this study there was heterogeneity within the CVI group in particular, with some tracts appearing similar to controls and others being substantially reduced in volume and number of fibers reconstructed.

## 5. Conclusions

This study investigated potential differences in tractography measures of white matter integrity in key pathways of the extended dorsal and ventral visual networks in those with CVI and controls. As a secondary exploratory analysis, the impact of CVI etiology was considered, specifically that of PVL compared to other causes. These results begin to provide empirical evidence for reduced white matter integrity in key pathways associated with the dorsal and ventral visual networks that may underlie the visual perceptual and visual function impairments observed in youths and young adults with CVI. The evidence reported as part of the exploratory analyses further indicate that individuals with PVL may demonstrate widespread changes to the extended visual processing networks. As such, it then becomes imperative that these children be monitored at regular intervals for potential visual and visual perceptual impairments frequently associated with CVI due to early developmental brain injury.

## Supplementary Material

supplemental_material

Supplementary material associated with this article can be found, in the online version, at https://doi.org/10.31083/j.jin2301001.

## Figures and Tables

**Table 1.** Demographic information for CVI and control participants.

Group	Participant	Age	Sex	Best-corrected visual acuity	CVI type	Etiology & comorbidities

CVI	1	19	female	20/20	CVI-nonPVL	seizure disorder
	2	17	female	20/70	CVI-nonPVL	infection
	3	17	female	20/40	CVI-PVL	PVL, CP (spastic diplegia)
	4	16	female	20/20	CVI-nonPVL	pre-eclampsia
	5	17	male	20/40	CVI-nonPVL	focal cortical atrophy, seizure disorder
	6	14	male	20/20	CVI-nonPVL	anoxia
	7	22	male	20/50	CVI-PVL	PVL, CP (spastic diplegia)
	8	14	male	20/75	CVI-nonPVL	neonatal infection
	9	17	male	unknown	CVI-PVL	PVL, CP (spastic diplegia)
	10	20	male	20/25	CVI-PVL	PVL, CP (spastic diplegia)

Control	1	21	female	20/20		
	2	17	female	20/20		
	3	22	female	20/20		
	4	15	female	20/20		
	5	24	female	20/20		
	6	15	female	20/20		
	7	17	female	20/20		
	8	17	female	20/20		
	9	24	female	20/20		
	10	22	female	20/20		
	11	23	female	20/20		
	12	23	female	20/20		
	13	19	male	20/20		
	14	18	male	20/20		
	15	18	male	20/20		
	16	25	male	20/20		
	17	17	male	20/20		

Note that acuity is best-corrected binocular visual acuity. Acuity and specification of etiology/comorbidities were obtained from participant medical records as provided by the participant and/or their caregiver. PVL, Periventricular leukomalacia; CP, cerebral palsy; CVI, cerebral visual impairment; CVI-PVL, CVI due to periventricular leukomalacia; CVI-nonPVL, CVI due to causes other than PVL.

**Table 2.** Mean volume of each tract for Control and CVI groups. Effects of age and group adjusted for age effects were compared with ANCOVA.

Tract	Control				CVI				Effect of Age			Effect of Group		

	Mean	SD	Min	Max	Mean	SD	Min	Max	Type III SS	F	*p*-value	Type III SS	F	*p*-value

L IFOF	32,929.8	16,484.25	9947	56,595	23,080.35	19,356.03	5573.75	68,808.2	8.38 × 10^8^	3.08	0.09	1.09 × 10^8^	0.40	0.53
L ILF	17,446.53	7748.94	3283	30,226.9	10,867.59	8752.74	1016.75	28,671.1	46,828,926	0.70	0.41	1.64 × 10^8^	2.46	0.13
L SLF	25,324.35	14,496.89	4305.88	48,001.6	20,214.33	15,364.24	361.38	42,072.6	5.85 × 10^8^	2.98	0.10	20,706,209	0.11	0.75
L VOF	4816.77	3056.58	655.38	10,706.5	4174.8	2189.65	1200.5	7539.88	24,779,335	3.94	0.06	63,191.67	0.01	0.92
R IFOF	30,611.68	14,903.57	9310	60,012.8	18,378.67	19,753.22	490	70,345.6	1.00 × 10^8^	0.36	0.55	7.21 × 10^8^	2.61	0.12
R ILF	18,738.89	8749.38	5292	32,021.5	13,358.02	9305.64	1292.38	33,546.6	77,226,270	1.02	0.32	1.11 × 10^8^	1.46	0.24
R SLF	28,497.1	15,503.24	3289.12	50,886.5	20,774.77	17,006.17	2229.5	48,975.5	5.42 × 10^8^	2.30	0.14	1.25 × 10^8^	0.53	0.47
R VOF	6418.28	2821.37	1335.25	11,882.5	6596.63	3461.62	2878.75	12,825.8	8,044,198	0.87	0.36	1,162,295	0.13	0.73

ANCOVA, analysis of covariance R, right; L, left; SD, standard deviation.

**Table 3.** Mean quantitative anisotropy of each tract for Control and CVI groups. Effects of age and group adjusted for age effects were compared with ANCOVA.

Tract	Control				CVI				Effect of age			Effect of group		

	Mean	SD	Min	Max	Mean	SD	Min	Max	Type III SS	F	*p*-value	Type III SS	F	*p*-value

L IFOF	0.27	0.07	0.16	0.45	0.30	0.08	0.19	0.47	0.0024	0.43	0.52	0.0024	0.44	0.51
L ILF	0.25	0.06	0.15	0.43	0.27	0.07	0.18	0.43	0.0067	1.59	0.22	0.0003	0.06	0.80
L SLF	0.23	0.07	0.14	0.39	0.28	0.09	0.18	0.49	0.0072	1.18	0.29	0.0049	0.80	0.38
L VOF	0.20	0.07	0.13	0.39	0.23	0.08	0.13	0.40	0.0122	2.35	0.14	0.0003	0.06	0.81
R IFOF	0.27	0.07	0.17	0.46	0.30	0.08	0.19	0.47	0.0096	1.72	0.20	0.0010	0.17	0.68
R ILF	0.24	0.07	0.15	0.43	0.27	0.08	0.18	0.45	0.0189	4.24	0.051	9.25 × 10^−6^	0	0.96
R SLF	0.24	0.07	0.14	0.42	0.28	0.07	0.19	0.41	0.0061	1.20	0.29	0.0031	0.60	0.45
R VOF	0.20	0.06	0.13	0.38	0.24	0.08	0.11	0.43	0.0116	2.38	0.14	0.0025	0.51	0.48

**Table 4.** Average mean diffusivity of each tract for Control and CVI groups. Effects of age and group adjusted for age effects were compared with ANCOVA.

Tract	Control				CVI				Effect of Age			Effect of Group		

	Mean	SD	Min	Max	Mean	SD	Min	Max	Type III SS	F	*p*-value	Type III SS	F	*p*-value

L IFOF	0.19	0.02	0.15	0.21	0.22	0.04	0.18	0.28	1.44 × 10^−5^	0.02	0.88	0.0057	8.86	0.007
L ILF	0.20	0.02	0.15	0.24	0.25	0.05	0.16	0.33	0.00079	0.60	0.45	0.0146	11.10	0.003
L SLF	0.12	0.02	0.09	0.18	0.15	0.03	0.11	0.20	1.60 × 10^−5^	0.02	0.89	0.0041	5.44	0.028
L VOF	0.18	0.02	0.14	0.22	0.21	0.03	0.18	0.29	3.64 × 10^−6^	0.01	0.94	0.0048	9.84	0.005
R IFOF	0.19	0.01	0.17	0.21	0.23	0.03	0.19	0.29	0.0006	1.28	0.27	0.0033	7.07	0.015
R ILF	0.20	0.02	0.16	0.26	0.24	0.05	0.19	0.35	0.0029	2.70	0.11	0.0007	11.85	0.0021
R SLF	0.12	0.02	0.09	0.16	0.16	0.04	0.10	0.22	0.0003	0.33	0.57	0.0067	8.44	0.0078
R VOF	0.18	0.03	0.12	0.26	0.20	0.03	0.16	0.25	0.0002	0.16	0.69	0.0107	23.76	<0.0001

**Table 5.** Mean axial diffusivity of each tract for Control and CVI groups. Effects of age and group adjusted for age effects were compared with ANCOVA.

Tract	Control				CVI				Effect of Age			Effect of Group		

	Mean	SD	Min	Max	Mean	SD	Min	Max	Type III SS	F	*p*-value	Type III SS	F	*p*-value

L IFOF	0.41	0.04	0.35	0.46	0.47	0.08	0.38	0.61	0.0007	0.25	0.62	0.0168	5.62	0.027
L ILF	0.43	0.04	0.34	0.49	0.50	0.08	0.36	0.60	0.0026	0.72	0.40	0.0337	9.34	0.005
L SLF	0.25	0.05	0.18	0.36	0.31	0.07	0.22	0.40	0.0000	0.01	0.94	0.0197	5.70	0.025
L VOF	0.35	0.04	0.26	0.41	0.39	0.04	0.34	0.47	0.0002	0.13	0.72	0.0078	5.43	0.029
R IFOF	0.41	0.03	0.37	0.47	0.47	0.05	0.42	0.56	0.0005	0.30	0.59	0.0256	16.95	0.0004
R ILF	0.41	0.04	0.33	0.49	0.46	0.06	0.40	0.59	0.0017	0.73	0.40	0.0223	9.31	0.006
R SLF	0.26	0.04	0.19	0.32	0.32	0.09	0.20	0.47	0.0016	0.44	0.52	0.0283	7.67	0.011
R VOF	0.34	0.05	0.25	0.42	0.37	0.05	0.32	0.48	0.0003	0.11	0.74	0.0061	2.54	0.124

**Table 6.** Mean radial diffusivity of each tract for Control and CVI groups. Effects of age and group adjusted for age effects were compared with ANCOVA.

Tract	Control				CVI				Effect of Age			Effect of Group		

	Mean	SD	Min	Max	Mean	SD	Min	Max	Type III SS	F	*p*-value	Type III SS	F	*p*-value

L IFOF	0.07	0.01	0.06	0.09	0.10	0.02	0.07	0.12	6.37 × 10^−5^	0.32	0.58	0.0023	11.51	0.003
L ILF	0.09	0.02	0.06	0.11	0.12	0.04	0.06	0.20	0.0003	0.36	0.56	0.0080	10.33	0.004
L SLF	0.05	0.01	0.04	0.09	0.07	0.02	0.05	0.10	6.78 × 10^−5^	0.36	0.56	0.0007	3.65	0.068
L VOF	0.09	0.02	0.07	0.12	0.12	0.03	0.09	0.19	9.62 × 10^−5^	0.19	0.67	0.0056	11.04	0.003
R IFOF	0.07	0.01	0.06	0.09	0.10	0.03	0.07	0.18	0.0006	2	0.17	0.0057	17.57	0.000
R ILF	0.09	0.02	0.07	0.15	0.13	0.04	0.08	0.22	0.0035	5.08	0.03	0.0088	12.61	0.002
R SLF	0.06	0.01	0.04	0.07	0.07	0.02	0.05	0.10	1.81 × 10^−5^	0.11	0.75	0.0015	9.02	0.006
R VOF	0.10	0.03	0.06	0.19	0.11	0.02	0.08	0.14	0.0001	0.18	0.68	0.0006	0.79	0.38

**Table 7.** Mean fractional diffusivity of each tract for Control and CVI groups. Effects of age and group adjusted for age effects were compared with ANCOVA.

Tract	Control				CVI				Effect of Age			Effect of Group		

	Mean	SD	Min	Max	Mean	SD	Min	Max	Type III SS	F	*p*-value	Type III SS	F	*p*-value

L IFOF	0.81	0.02	0.76	0.83	0.78	0.03	0.74	0.83	0.0012	2.06	0.16	0.0020	3.41	0.078
L ILF	0.79	0.03	0.75	0.83	0.75	0.05	0.67	0.81	0.0000033	0	0.96	0.0084	6.26	0.020
L SLF	0.79	0.02	0.75	0.82	0.78	0.03	0.72	0.82	0.0004	0.75	0.40	0.0001	0.20	0.660
L VOF	0.73	0.03	0.68	0.77	0.69	0.05	0.60	0.74	0.0004	0.27	0.61	0.0135	9.91	0.004
R IFOF	0.80	0.02	0.77	0.83	0.77	0.05	0.64	0.82	0.0019	1.63	0.21	0.0094	8.07	0.009
R ILF	0.76	0.03	0.68	0.81	0.72	0.05	0.62	0.79	0.0088	7.47	0.01	0.0133	11.31	0.003
R SLF	0.78	0.01	0.77	0.81	0.77	0.02	0.73	0.80	0.000037	0.14	0.71	0.0005	1.99	0.170
R VOF	0.70	0.06	0.51	0.77	0.70	0.04	0.62	0.75	0.0012	0.39	0.54	0.000024	0.01	0.930

## Data Availability

Data are available upon request and in alignment with IRB of Massachusetts Eye and Ear (MEE).

## Abbreviations

CVI: cerebral visual impairment
PVL: perivnetricualr leukomalacia
CVI-PVL: Cerebral visual impairment due to periventricular leukomalacia
CVI-nonPVL: cerebral visual impairment not due to periventricular leukomalacia
SLF: superior longitudinal fasciculus
ILF: inferior longitudinal fasciculus
IFOF: inferior fronto-occipital fasciculus
VOF: verical occipital fasciculus
FA: fractional anisotropy
QA: quantitative anisotropy
MD: mean diffusivity
AD: axial diffusivity
RD: radial diffusivity
ICV: intracranial volume
R: right
L: left
ANCOVA: analysis of covariance
ANOVA: analysis of variance
SD: standard deviation
HARDI: high angular resolution diffusion imaging
ODF: orientation distribution function
TE: echo time
TR: relaxation time
MRI: magnetic resonance imaging
