# Supplementary material for: Aberrant White Matter Development in Cerebral Visual Impairment: A Proposed Mechanism for Visual Dysfunction Following Early Brain Injury
Source: J Integr Neurosci. Author manuscript; Available in PMC 2025 Sep 15. (PMC12434583; doi:10.31083/j.jin2301001)
Supplement: supplemental_material [file NIHMS2107070-supplement-supplemental_material.docx]

**
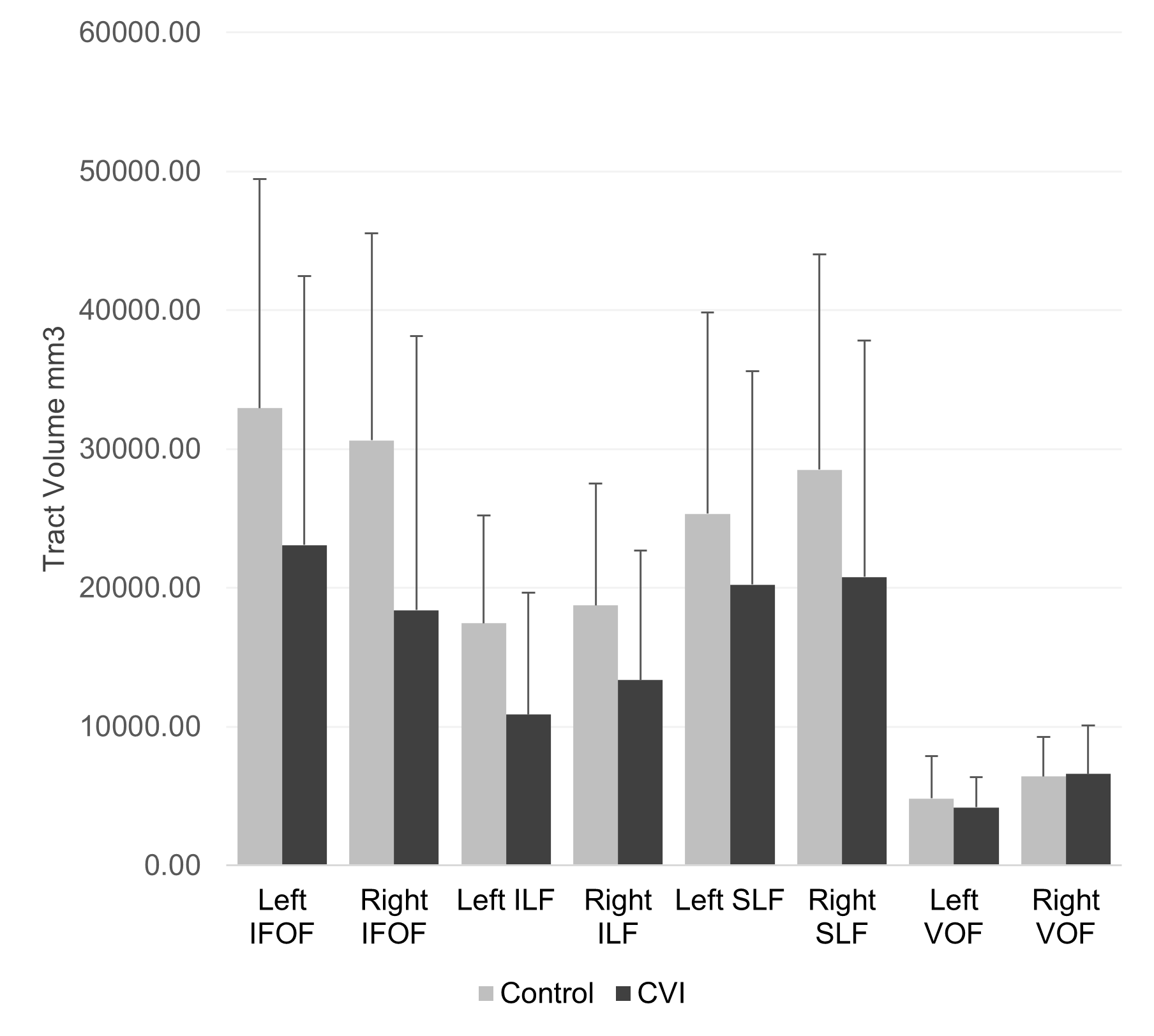
**

Supplementary Fig. 1. Group comparisons in tract volume adjusted for age (ANCOVA). Note: raw data shown for ease of visualization, but for statistical analyses, adjusted for intracranial volume using residuals. Although volumes appear to be reduced in C


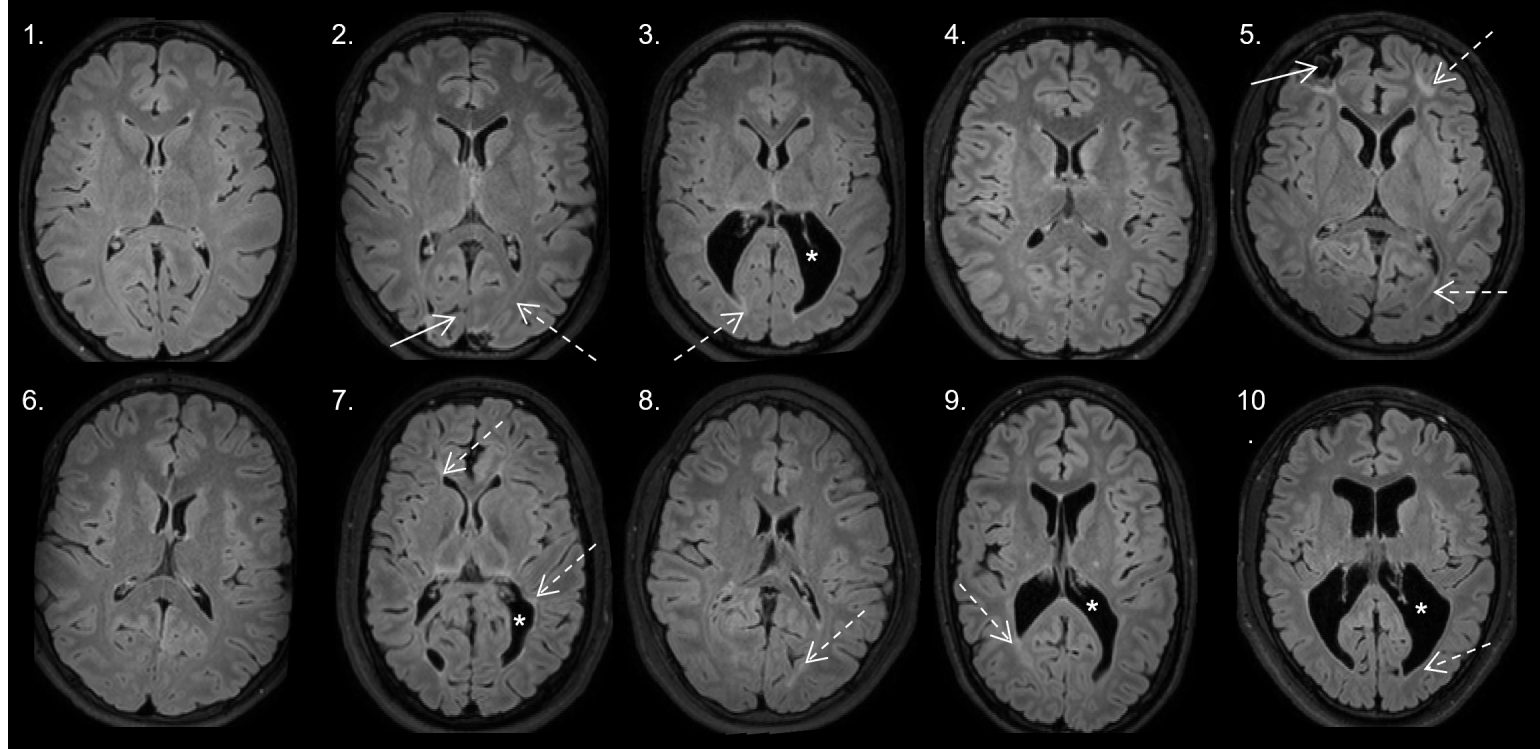


Supplementary Fig. 2. Axial FLAIR for each of the participants with CVI. The numbers correspond to the participants in Table 1. Note the heterogeneous extent and distribution of white matter lesions (hyperintensities – examples are indicated by dashed arrows), varying degrees of ventriculomegaly (e.g., presence of large lateral ventricles particularly seen in participants 3, 7, 9, and 10 – indicated by asterisks, as well as the presence of cortical abnormalities in participants 2 and 5 (indicated by solid white arrow).

|  |  | **Control** | | | | **CVI** | | | | **Effect of Age** | | | **Effect of Group** | | |
| --- | --- | --- | --- | --- | --- | --- | --- | --- | --- | --- | --- | --- | --- | --- | --- |
| **QA** | **Tract** | **Mean** | **S.D.** | **Min** | **Max** | **Mean** | **S.D.** | **Min** | **Max** | **Type III SS** | **F** | **p-value** | **Type III SS** | **F** | **p-value** |
|  | L SLF I | 0.23 | 0.08 | 0.13 | 0.39 | 0.27 | 0.10 | 0.15 | 0.49 | 0.007133 | 0.97 | 0.3337 | 0.004361 | 0.6 | 0.448 |
|  | L SLF II | 0.22 | 0.05 | 0.15 | 0.31 | 0.27 | 0.10 | 0.18 | 0.49 | 2.8E-07 | 0 | 0.9943 | 0.010418 | 1.94 | 0.1782 |
|  | L SLF III | 0.20 | 0.05 | 0.14 | 0.29 | 0.27 | 0.10 | 0.18 | 0.49 | 4.77E-05 | 0.01 | 0.9261 | 0.016931 | 3.14 | 0.0919 |
|  | R SLF I | 0.24 | 0.07 | 0.14 | 0.41 | 0.27 | 0.07 | 0.18 | 0.40 | 0.00442 | 0.87 | 0.3599 | 0.001586 | 0.31 | 0.5813 |
|  | R SLF II | 0.25 | 0.07 | 0.14 | 0.44 | 0.28 | 0.10 | 0.20 | 0.47 | 0.004684 | 0.74 | 0.3998 | 0.00086 | 0.14 | 0.7164 |
|  | R SLF III | 0.21 | 0.05 | 0.14 | 0.30 | 0.27 | 0.09 | 0.18 | 0.45 | 0.000145 | 0.03 | 0.8656 | 0.013552 | 2.73 | 0.1125 |
| **MD** | **Tract** | **Mean** | **S.D.** | **Min** | **Max** | **Mean** | **S.D.** | **Min** | **Max** | **Type III SS** | **F** | **p-value** | **Type III SS** | **F** | **p-value** |
|  | L SLF I | 0.14 | 0.02 | 0.11 | 0.18 | 0.17 | 0.02 | 0.14 | 0.21 | 8.49E-05 | 0.17 | 0.6814 | 0.00484 | 9.84 | 0.0045 |
|  | L SLF II | 0.11 | 0.02 | 0.07 | 0.14 | 0.13 | 0.03 | 0.09 | 0.17 | 0.0006 | 1.3 | 0.2674 | 0.003268 | 7.07 | 0.0147 |
|  | L SLF III | 0.12 | 0.02 | 0.09 | 0.15 | 0.14 | 0.03 | 0.10 | 0.20 | 0.000371 | 0.6 | 0.4483 | 0.000728 | 1.17 | 0.2916 |
|  | R SLF I | 0.14 | 0.02 | 0.10 | 0.17 | 0.17 | 0.03 | 0.12 | 0.23 | 0.00024 | 0.39 | 0.5395 | 0.004024 | 6.5 | 0.0176 |
|  | R SLF II | 0.11 | 0.02 | 0.09 | 0.15 | 0.12 | 0.02 | 0.09 | 0.15 | 2E-08 | 0 | 0.9947 | 0.000382 | 1 | 0.3277 |
|  | R SLF III | 0.13 | 0.02 | 0.09 | 0.17 | 0.15 | 0.02 | 0.12 | 0.18 | 0.00036 | 0.82 | 0.374 | 0.004631 | 10.6 | 0.0036 |
| **AD** | **Tract** | **Mean** | **S.D.** | **Min** | **Max** | **Mean** | **S.D.** | **Min** | **Max** | **Type III SS** | **F** | **p-value** | **Type III SS** | **F** | **p-value** |
|  | L SLF I | 0.29 | 0.05 | 0.22 | 0.37 | 0.36 | 0.06 | 0.28 | 0.47 | 8.01E-05 | 0.03 | 0.8635 | 0.021834 | 8.23 | 0.0085 |
|  | L SLF II | 0.22 | 0.03 | 0.15 | 0.30 | 0.27 | 0.07 | 0.19 | 0.37 | 0.00292 | 1.34 | 0.2607 | 0.014017 | 6.41 | 0.0194 |
|  | L SLF III | 0.25 | 0.04 | 0.19 | 0.30 | 0.30 | 0.06 | 0.22 | 0.41 | 0.000984 | 0.45 | 0.5118 | 0.003683 | 1.67 | 0.2111 |
|  | R SLF I | 0.30 | 0.04 | 0.21 | 0.36 | 0.34 | 0.07 | 0.25 | 0.48 | 0.001959 | 0.68 | 0.4181 | 0.017186 | 5.96 | 0.0224 |
|  | R SLF II | 0.23 | 0.03 | 0.18 | 0.30 | 0.25 | 0.05 | 0.18 | 0.31 | 0.000108 | 0.07 | 0.7994 | 0.000528 | 0.32 | 0.5761 |
|  | R SLF III | 0.26 | 0.04 | 0.19 | 0.36 | 0.31 | 0.05 | 0.25 | 0.41 | 0.002301 | 1.15 | 0.2953 | 0.019712 | 9.85 | 0.0048 |
| **RD** | **Tract** | **Mean** | **S.D.** | **Min** | **Max** | **Mean** | **S.D.** | **Min** | **Max** | **Type III SS** | **F** | **p-value** | **Type III SS** | **F** | **p-value** |
|  | L SLF I | 0.07 | 0.01 | 0.05 | 0.09 | 0.08 | 0.02 | 0.06 | 0.12 | 8.74E-05 | 0.53 | 0.4753 | 0.000929 | 5.59 | 0.0265 |
|  | L SLF II | 0.05 | 0.01 | 0.03 | 0.07 | 0.06 | 0.02 | 0.04 | 0.10 | 9.47E-05 | 0.7 | 0.4131 | 0.000705 | 5.19 | 0.0333 |
|  | L SLF III | 0.06 | 0.01 | 0.04 | 0.08 | 0.07 | 0.02 | 0.04 | 0.09 | 0.000174 | 0.79 | 0.3853 | 0.000103 | 0.46 | 0.5041 |
|  | R SLF I | 0.07 | 0.01 | 0.05 | 0.09 | 0.08 | 0.01 | 0.06 | 0.10 | 1.24E-06 | 0.01 | 0.927 | 0.000877 | 6.04 | 0.0216 |
|  | R SLF II | 0.05 | 0.01 | 0.04 | 0.07 | 0.06 | 0.01 | 0.04 | 0.07 | 2.51E-05 | 0.26 | 0.6139 | 0.000318 | 3.32 | 0.0826 |
|  | R SLF III | 0.06 | 0.01 | 0.04 | 0.08 | 0.07 | 0.01 | 0.05 | 0.09 | 1.99E-05 | 0.16 | 0.6972 | 0.001017 | 7.94 | 0.01 |
| **FA** | **Tract** | **Mean** | **S.D.** | **Min** | **Max** | **Mean** | **S.D.** | **Min** | **Max** | **Type III SS** | **F** | **p-value** | **Type III SS** | **F** | **p-value** |
|  | L SLF I | 0.78 | 0.02 | 0.74 | 0.80 | 0.76 | 0.05 | 0.65 | 0.82 | 0.000317 | 0.28 | 0.6035 | 0.000436 | 0.38 | 0.5429 |
|  | L SLF II | 0.80 | 0.02 | 0.77 | 0.82 | 0.79 | 0.03 | 0.72 | 0.82 | 5.57E-05 | 0.11 | 0.7436 | 0.00034 | 0.67 | 0.4221 |
|  | L SLF III | 0.79 | 0.01 | 0.76 | 0.81 | 0.78 | 0.04 | 0.74 | 0.83 | 0.000813 | 1.39 | 0.252 | 0.000197 | 0.34 | 0.5675 |
|  | R SLF I | 0.78 | 0.02 | 0.74 | 0.81 | 0.76 | 0.02 | 0.73 | 0.80 | 0.000241 | 0.69 | 0.413 | 0.000375 | 1.08 | 0.3089 |
|  | R SLF II | 0.80 | 0.02 | 0.76 | 0.83 | 0.78 | 0.01 | 0.76 | 0.80 | 0.000459 | 1.55 | 0.2265 | 0.00113 | 3.82 | 0.064 |
|  | R SLF III | 0.78 | 0.01 | 0.75 | 0.81 | 0.77 | 0.03 | 0.73 | 0.81 | 0.000224 | 0.52 | 0.4771 | 0.000163 | 0.38 | 0.543 |
| **Volume** | **Tract** | **Mean** | **S.D.** | **Min** | **Max** | **Mean** | **S.D.** | **Min** | **Max** | **Type III SS** | **F** | **p-value** | **Type III SS** | **F** | **p-value** |
|  | L SLF I | 10864.31 | 8405.00 | 3515.75 | 32156.20 | 7924.52 | 6501.81 | 361.38 | 19195.80 | 1.84E+08 | 3.51 | 0.073 | 8057216 | 0.15 | 0.6985 |
|  | L SLF II | 11975.53 | 6625.18 | 2529.62 | 24371.40 | 10735.59 | 5737.84 | 3166.62 | 18326.00 | 84094964 | 2.28 | 0.1456 | 619586 | 0.02 | 0.898 |
|  | L SLF III | 7186.66 | 4585.94 | 275.63 | 17125.50 | 7412.02 | 5660.20 | 643.13 | 16947.90 | 69727253 | 3.34 | 0.0826 | 19514773 | 0.93 | 0.3452 |
|  | R SLF I | 8992.95 | 5236.16 | 1684.38 | 16065.90 | 7432.07 | 5924.55 | 1610.88 | 20102.20 | 43564456 | 1.59 | 0.2199 | 4971057 | 0.18 | 0.6742 |
|  | R SLF II | 12474.46 | 8006.20 | 496.13 | 28260.80 | 10791.39 | 7983.16 | 410.38 | 20469.80 | 3.43E+08 | 7.06 | 0.0147 | 18531866 | 0.38 | 0.5433 |
|  | R SLF III | 12301.04 | 4887.82 | 3105.38 | 20292.10 | 8816.31 | 7251.39 | 618.63 | 19348.90 | 1055439 | 0.03 | 0.8604 | 69442769 | 2.08 | 0.1631 |

**Supplementary Table 1.** Group means and ANCOVA results for the three subdivisions of the SLF in control and CVI groups. Group comparisons are adjusted for the potential effects of age.

|  | | **Age** | | | **CVI group** | | |
| --- | --- | --- | --- | --- | --- | --- | --- |
| **Volume** | **Tract** | **Type III SS** | **F** | **p** | **Type III SS** | **F** | **p** |
|  | L IFOF | 1.14E+09 | 4.71 | 0.0411 | 1.01E+09 | 2.07 | 0.1502 |
|  | L ILF | 1.4E+08 | 2.56 | 0.1234 | 5.02E+08 | 4.59 | 0.021 |
|  | L SLF | 9.7E+08 | 5.65 | 0.0262 | 7.93E+08 | 2.31 | 0.122 |
|  | L VOF | 24305794 | 3.71 | 0.0664 | 378845.6 | 0.03 | 0.9715 |
|  | R IFOF | 3.61E+08 | 1.5 | 0.2335 | 1.8E+09 | 3.73 | 0.0394 |
|  | R ILF | 2.07E+08 | 3.42 | 0.0774 | 5.42E+08 | 4.49 | 0.0226 |
|  | R SLF | 1.07E+09 | 5.64 | 0.0263 | 1.43E+09 | 3.78 | 0.038 |
|  | R VOF | 10514683 | 1.11 | 0.3036 | 4522862 | 0.24 | 0.79 |

**Supplementary Table 2** ANCOVA results for comparisons of tract volume between control, CVI-PVL, and CVI-nonPVL groups adjusting for the potential effects of age.

|  | | **Age** | | | **CVI group** | | |
| --- | --- | --- | --- | --- | --- | --- | --- |
| **QA** | **Tract** | **Type III SS** | **F** | **p** | **Type III SS** | **F** | **p** |
|  | L IFOF | 0.004284 | 0.82 | 0.3762 | 0.012151 | 1.16 | 0.3329 |
|  | L ILF | 0.010347 | 2.53 | 0.1254 | 0.006992 | 0.85 | 0.4385 |
|  | L SLF | 0.015028 | 2.72 | 0.1127 | 0.025429 | 2.3 | 0.1227 |
|  | L VOF | 0.019191 | 3.96 | 0.0586 | 0.013308 | 1.37 | 0.2734 |
|  | R IFOF | 0.012684 | 2.25 | 0.1471 | 0.00521 | 0.46 | 0.6355 |
|  | R ILF | 0.02376 | 5.43 | 0.029 | 0.006193 | 0.71 | 0.5035 |
|  | R SLF | 0.010987 | 2.27 | 0.1456 | 0.01402 | 1.45 | 0.2558 |
|  | R VOF | 0.020737 | 4.91 | 0.0369 | 0.022568 | 2.67 | 0.0906 |

**Supplementary Table 3.** ANCOVA results for comparisons of QA between control, CVI-PVL, and CVI-nonPVL groups adjusting for the potential effects of age.

|  | | **Age** | | | **CVI group** | | |
| --- | --- | --- | --- | --- | --- | --- | --- |
| **AD** | **Tract** | **Type III SS** | **F** | **p** | **Type III SS** | **F** | **p** |
|  | L IFOF | 7.48E-05 | 0.03 | 0.868 | 0.027194 | 5.14 | 0.0147 |
|  | L ILF | 0.000166 | 0.05 | 0.8184 | 0.049412 | 8.01 | 0.0023 |
|  | L SLF | 0.000801 | 0.26 | 0.6134 | 0.032644 | 5.35 | 0.0124 |
|  | L VOF | 9.32E-05 | 0.06 | 0.8048 | 0.007956 | 2.67 | 0.0908 |
|  | R IFOF | 7.19E-05 | 0.06 | 0.8032 | 0.03582 | 15.84 | <.0001 |
|  | R ILF | 0.000698 | 0.29 | 0.5947 | 0.024509 | 5.11 | 0.0146 |
|  | R SLF | 5.15E-05 | 0.02 | 0.8924 | 0.0537 | 9.76 | 0.0009 |
|  | R VOF | 0.000655 | 0.27 | 0.609 | 0.007287 | 1.49 | 0.2453 |

**Supplementary Table 4** ANCOVA results for comparisons of AD between control, CVI-PVL, and CVI-nonPVL groups adjusting for the potential effects of age.

|  | | **Age** | | | **CVI group** | | |
| --- | --- | --- | --- | --- | --- | --- | --- |
| **RD** | **Tract** | **Type III SS** | **F** | **p** | **Type III SS** | **F** | **p** |
|  | L IFOF | 0.000152 | 0.84 | 0.3698 | 0.002929 | 8.09 | 0.0023 |
|  | L ILF | 1.8E-06 | 0 | 0.96 | 0.010656 | 7.62 | 0.0029 |
|  | L SLF | 6.11E-05 | 0.31 | 0.5839 | 0.000693 | 1.75 | 0.1961 |
|  | L VOF | 0.000224 | 0.44 | 0.5154 | 0.005969 | 5.83 | 0.009 |
|  | R IFOF | 0.00032 | 1.02 | 0.3239 | 0.006167 | 9.8 | 0.0008 |
|  | R ILF | 0.002403 | 3.46 | 0.0758 | 0.009595 | 6.9 | 0.0045 |
|  | R SLF | 3.37E-06 | 0.02 | 0.8849 | 0.001948 | 6.18 | 0.0071 |
|  | R VOF | 7.57E-05 | 0.1 | 0.7574 | 0.00065 | 0.42 | 0.6622 |

**Supplementary Table 5** ANCOVA results for comparisons of RD between control, CVI-PVL, and CVI-nonPVL groups adjusting for the potential effects of age.

|  | | **Age** | | | **CVI group** | | |
| --- | --- | --- | --- | --- | --- | --- | --- |
| **MD** | **Tract** | **Type III SS** | **F** | **p** | **Type III SS** | **F** | **p** |
|  | L IFOF | 2.84E-05 | 0.05 | 0.8223 | 0.00822 | 7.47 | 0.0033 |
|  | L ILF | 2.7E-05 | 0.02 | 0.8784 | 0.020391 | 9.06 | 0.0013 |
|  | L SLF | 0.000214 | 0.29 | 0.5939 | 0.005566 | 3.79 | 0.0376 |
|  | L VOF | 4.56E-05 | 0.07 | 0.7986 | 0.006581 | 4.82 | 0.0179 |
|  | R IFOF | 8.27E-05 | 0.23 | 0.6397 | 0.013079 | 17.79 | <.0001 |
|  | R ILF | 0.001721 | 1.63 | 0.2151 | 0.013802 | 6.52 | 0.0057 |
|  | R SLF | 1.31E-05 | 0.02 | 0.8875 | 0.011211 | 8.76 | 0.0015 |
|  | R VOF | 0.000205 | 0.19 | 0.6692 | 0.001815 | 0.83 | 0.4497 |

**Supplementary Table 6** ANCOVA results for comparisons of MD between control, CVI-PVL, and CVI-nonPVL groups adjusting for the potential effects of age.

|  | | **Age** | | | **CVI group** | | |
| --- | --- | --- | --- | --- | --- | --- | --- |
| **FA** | **Tract** | **Type III SS** | **F** | **p** | **Type III SS** | **F** | **p** |
|  | L IFOF | 0.001168 | 1.91 | 0.1812 | 0.001997 | 1.63 | 0.2186 |
|  | L ILF | 9.97E-05 | 0.07 | 0.7894 | 0.009228 | 3.38 | 0.0517 |
|  | L SLF | 4.49E-05 | 0.11 | 0.7484 | 0.001649 | 1.94 | 0.1671 |
|  | L VOF | 0.000626 | 0.45 | 0.5101 | 0.014035 | 5.02 | 0.0155 |
|  | R IFOF | 0.001453 | 1.2 | 0.284 | 0.009552 | 3.96 | 0.0334 |
|  | R ILF | 0.006796 | 5.68 | 0.0258 | 0.013919 | 5.82 | 0.009 |
|  | R SLF | 4.58E-06 | 0.02 | 0.8965 | 0.000683 | 1.29 | 0.2944 |
|  | R VOF | 0.000524 | 0.16 | 0.6904 | 0.00143 | 0.22 | 0.8027 |

**Supplementary Table 7** ANCOVA results for comparisons of FA between control, CVI-PVL, and CVI-nonPVL groups adjusting for the potential effects of age.

|  | | **Age** | | | **CVI type** | | |
| --- | --- | --- | --- | --- | --- | --- | --- |
| **Imaging Outcome** | **Tract** | **Type III SS** | **F** | **p** | **Type III SS** | **F** | **p** |
| AD | L SLF I | 0.001941 | 0.92 | 0.3466 | 0.037159 | 8.84 | 0.0014 |
|  | L SLF II | 0.001441 | 0.84 | 0.3705 | 0.025584 | 7.45 | 0.0038 |
|  | L SLF III | 0.000939 | 0.4 | 0.5322 | 0.003746 | 0.81 | 0.4606 |
|  | R SLF I | 4.51E-05 | 0.02 | 0.8907 | 0.032689 | 6.99 | 0.0042 |
|  | R SLF II | 0.000162 | 0.11 | 0.7451 | 0.00507 | 1.7 | 0.208 |
|  | R SLF III | 0.000757 | 0.39 | 0.5385 | 0.023097 | 5.96 | 0.0089 |
| FA | L SLF I | 3.87E-05 | 0.03 | 0.855 | 0.001865 | 0.82 | 0.4511 |
|  | L SLF II | 0.000184 | 0.42 | 0.526 | 0.002168 | 2.46 | 0.111 |
|  | L SLF III | 0.000602 | 1.23 | 0.2816 | 0.002574 | 2.63 | 0.0983 |
|  | R SLF I | 0.000157 | 0.44 | 0.5148 | 0.000441 | 0.61 | 0.5498 |
|  | R SLF II | 0.000459 | 1.48 | 0.2378 | 0.001131 | 1.82 | 0.1875 |
|  | R SLF III | 3.13E-05 | 0.08 | 0.7854 | 0.000933 | 1.13 | 0.3406 |
| MD | L SLF I | 0.000454 | 1.05 | 0.3151 | 0.006737 | 7.82 | 0.0026 |
|  | L SLF II | 0.000372 | 0.87 | 0.3615 | 0.00445 | 5.22 | 0.015 |
|  | L SLF III | 0.000321 | 0.5 | 0.487 | 0.000982 | 0.77 | 0.4781 |
|  | R SLF I | 4.1E-07 | 0 | 0.9778 | 0.006944 | 6.68 | 0.0051 |
|  | R SLF II | 1.28E-06 | 0 | 0.9528 | 0.001225 | 1.71 | 0.2055 |
|  | R SLF III | 0.000149 | 0.34 | 0.5667 | 0.00498 | 5.65 | 0.0109 |
| QA | L SLF I | 0.012814 | 1.81 | 0.1921 | 0.017052 | 1.2 | 0.3189 |
|  | L SLF II | 0.000674 | 0.17 | 0.6839 | 0.044148 | 5.59 | 0.0118 |
|  | L SLF III | 7.63E-05 | 0.02 | 0.8869 | 0.055152 | 7.51 | 0.004 |
|  | R SLF I | 0.007268 | 1.44 | 0.2424 | 0.007244 | 0.72 | 0.4985 |
|  | R SLF II | 0.005395 | 0.97 | 0.3374 | 0.022302 | 2 | 0.162 |
|  | R SLF III | 0.002127 | 0.46 | 0.5042 | 0.025984 | 2.82 | 0.0821 |
| RD | L SLF I | 9.88E-05 | 0.57 | 0.4574 | 0.00094 | 2.72 | 0.0871 |
|  | L SLF II | 9.89E-05 | 0.69 | 0.4143 | 0.00071 | 2.49 | 0.108 |
|  | L SLF III | 0.000134 | 0.63 | 0.4367 | 0.000499 | 1.18 | 0.3298 |
|  | R SLF I | 1.86E-05 | 0.14 | 0.7147 | 0.00123 | 4.52 | 0.0221 |
|  | R SLF II | 2.18E-05 | 0.23 | 0.6384 | 0.000415 | 2.17 | 0.1401 |
|  | R SLF III | 2.1E-05 | 0.16 | 0.6966 | 0.001018 | 3.79 | 0.0392 |
| Volume | L SLF I | 2.63E+08 | 5.35 | 0.0301 | 1.34E+08 | 1.36 | 0.2762 |
|  | L SLF II | 1.09E+08 | 3.19 | 0.0895 | 89875129 | 1.31 | 0.291 |
|  | L SLF III | 66148380 | 3.05 | 0.0968 | 25196486 | 0.58 | 0.5689 |
|  | R SLF I | 78705819 | 3.12 | 0.0905 | 84024533 | 1.67 | 0.2109 |
|  | R SLF II | 3.53E+08 | 7.4 | 0.0132 | 84092038 | 0.88 | 0.4295 |
|  | R SLF III | 20270135 | 0.7 | 0.411 | 1.98E+08 | 3.44 | 0.051 |

**Supplementary Table 8.** ANCOVA results for comparisons of tract outcomes for the SLF subdivisions between control, CVI-PVL, and CVI-nonPVL groups adjusting for the potential effects of age.
